# Grover's Disease after Heart Transplantation: A Case Report

**DOI:** 10.1155/2012/126592

**Published:** 2012-12-20

**Authors:** Giovanbattista Ippoliti, Marco Paulli, Marco Lucioni, Andrea Maria D'Armini, Marinella Lauriola, Rany Mahrous Haleem Saaleb

**Affiliations:** ^1^Division of Cardiac Surgery, IRCCS San Matteo Hospital Foundation, University of Pavia School of Medicine, 27100 Pavia, Italy; ^2^U.O. Medicina Interna, Policlinico di Monza, Monza, Italy; ^3^Anatomic Pathology, IRCCS San Matteo Hospital Foundation, University of Pavia, 27100 Pavia, Italy

## Abstract

Grover's disease is a transient acantholytic dermatosis of unknown cause, manifesting clinically as a papular skin eruption that is usually located on the anterior chest and abdomen. Histologically characterized by an acantholytic pattern, it has been associated with numerous disorders, including hematologic malignancies, chronic renal failure, and HIV infection, as well as with chemotherapy and bone marrow and/or kidney transplant. Evaluation of followup and treatment is often complicated by spontaneous remission and the occasionally fluctuant course of the disease. Here we report the case of a patient with sudden onset of Grover's disease after heart transplantation. To the best of our knowledge, this is the first observation of Grover's disease as diagnosed after heart transplantation.

## 1. Introduction

After organ transplantation, patients with immunosuppressive therapy are at risk from various skin diseases that are related to infections or malignancies [1]. Grover's disease (GD), also named “transient acantholytic dermatosis,” is an unusual disease that mostly affects middle-aged and elderly white men; its etiology is still largely unknown.

Patients develop pruriginous skin papules, papulovesicles, and small nodules, mainly on the chest, back, lumbar area, and extremities [2, 3]. The histological diagnosis of clinically suspected lesions may be compromised by GD's capacity to adopt differing patterns, and by the fact that involved areas are generally diagnosed as focal. The histopathologic hallmark of the disease is acantholysis, frequently in combination with dyskeratosis, with the result that the lesions resemble those of Darier disease, Hailey-Hailey disease, or pemphigus [4].

We here report, to the best of our knowledge, the first case of histologically confirmed GD to occur in a heart transplanted patient.

## 2. Case Report

**Figure 1:**
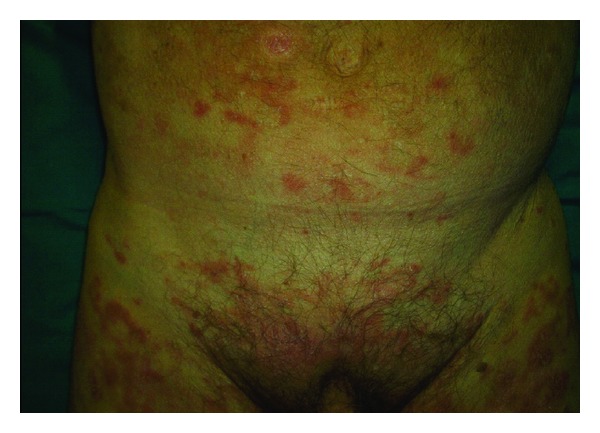
Extensive eruption of erythematous red-brown papulovesicles lesions scattered over the abdomen and the lower extremities.

A 64-year-old man was hospitalized at our institution with a one-month history of mildly pruritic skin lesions located on the abdomen and the lower extremities. Physical examination revealed numerous erythematous red-brown papulovesicles (Figure 1).

**Figure 2:**
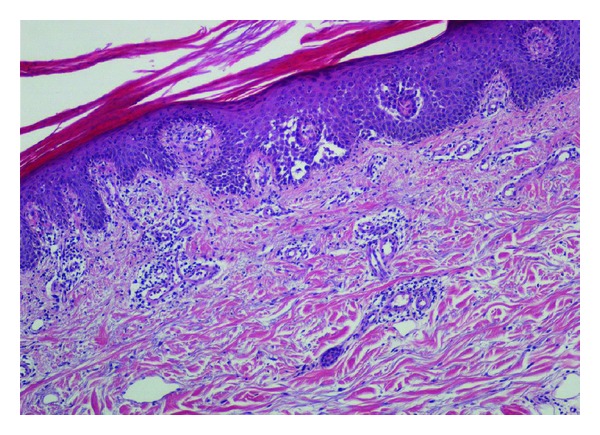
Skin biopsy showing foci of acantholytic dermatosis and loose perivascular lymphoid infiltrate in the superficial dermis (H&E 100x).

**Figure 3:**
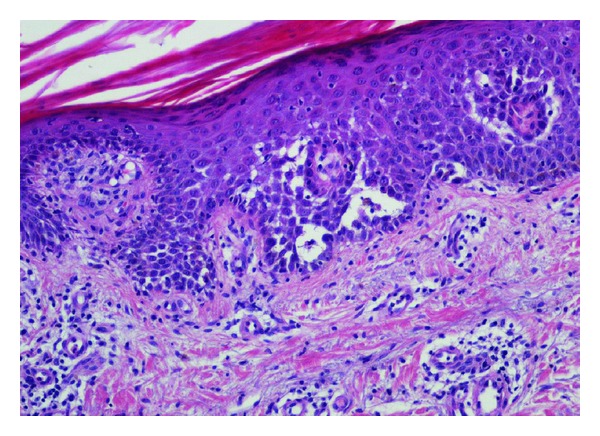
Epidermis showed hypergranulosis and suprabasal clefts containing acantholytic dyskeratotic keratinocytes (H&E 200x).

The patient had undergone heart transplantation (HTx) 22 years previously on account of dilatated cardiomyopathy. After HTx, immunosuppression consisted of RATG (Rabbit antithymocyte globulin) for three days, cyclosporine A (CSA), azathioprine, and steroids. No acute rejection episodes were observed during followup. Eleven years after HTx, the patient developed a posttransplant lymphoproliferative disorder (PTLD) which, at biopsy, proved to be an EBV-negative B-cell diffuse large cell lymphoma (B-DLCL). Azathioprine was eliminated and CSA doses were reduced by 50%. Chemotherapy with CHOP regimen (cyclophosphamide, vincristine, adriblastine, and prednisone) was started and continued for a total of six treatment cycles. Complete remission was achieved and confirmed by continuous followup. On admission to our center, the patient was on low-doses of CSA (trough levels: 70 *μ*g/L) and prednisone (2.5 mg/d). Laboratory data revealed creatinine = 2.67 mg/dL. Autoimmunity, cryoglobulins, and PCR for EBV, CMV, HHV-8, HCV, and Parvovirus B19 were negative. No relapse of NHL was observed. Punch biopsies from two different cutaneous lesions were performed and histological examination of both skin samples documented an acantholytic dermatosis, which exhibited a Darier disease-like pattern. Focal hypergranulosis and suprabasal clefts containing acantholytic and dyskeratotic keratinocytes were observed in the epidermis. A loose perivascular lymphoid infiltrate, which included a few eosinophils and neutrophils, was found in the superficial dermis (Figures 2 and 3). Direct immunofluorescence was negative. During hospitalization, skin lesions began to improve. The patient was discharged to the outpatient-clinic to continue follow up. Skin lesions disappeared in two months. Clinicopathologic features were consistent with the diagnosis of transient acantholytic dermatosis (GD).

## 3. Discussion

The etiology of GD is still unclear, but many factors have been associated with the development of the disease. Authors have variously linked the disorder with prolonged fever, prolonged bed rest, excessive heat, and profuse sweating [5]. The latter two features are assumed to derive from sweat ducts obstruction and from the leakage of products that induce acantholysis [6]. However, the possibility still exists that smaller molecules may seep through the ductal epithelium and cause epidermal acantholysis [7]. In addition, heat may directly cause acantholysis through the release of an unknown enzymatic factor after mild thermal injury [8].

Solar damage is a common association [9], but GD has also been associated with drugs abuse, various malignancies, immunodeficiency conditions, including HIV infection, and bone marrow transplantation [5, 10].

GD was observed during chemotherapy treatment for neoplasia, especially after etoposide and ARA-C administration [11, 12]. Quite recently, a case of cetuximab-induced GD was reported in an oncologic patient [13]. Our patient received chemotherapy eleven years prior GD onset and has recorded no NHL relapse to date. No correlation whatsoever may be deduced between GD and haematological disorder or chemotherapy.

The incidence of GD in marrow transplant patients is about 1.8% [14], while a single exclusive case has been reported as occurring after renal transplantation [15].

Our patient is accordingly the first reported case of GD developing in a heart transplant recipient under immunosuppressive therapy. Although this association could be fortuitous, the fact that GD often occurs in immunosuppressed patients suggests that immunosuppression could be a factor that favors the development of GD [15].

Recently the histologic spectrum of GD has been widened from its classic 4-pattern basis (Darier disease-like, Hailey-Hailey disease-like, pemphigus-like, and spongiotic) to include some newly identified patterns [4]. Unawareness of these unusual features may impede diagnosis as GD or lead to outright-misdiagnosis as actinic keratosis, solar keratosis, insect bites reactions or drug eruptions. Given the foregoing, we cannot exclude that the incidence of posttransplant GD may have been underestimated too.

Despite scrupulous examination of the patient, we could not identify a clear-cut relation of disease onset with any of the factors known to be associated with GD. The only anamnestic finding consisted in a three-day flu-induced bed rest, which occurred one month prior to skin eruption. We cannot exclude that prolonged immunosuppressive therapy may itself represent an additional factor that elicits GD onset.

Although GD is generally self-limited and resolves within weeks or months even in immunocompromised patients [16], in some cases it may persist or recur [3]. Sunlight, excessive heat and profuse sweating must be avoided. Topical therapy consists of steroids, tretinoin, and calcipotriol. Systemic therapy includes oral vitamine A, corticosteroids, synthetic retinoids, and psoralen-PUVA [17].

This report expands the list of differential diagnosis considerations for cutaneous eruption in HTx patients. Although self-limited, Grover's disease must be considered in the differential diagnosis of cutaneous eruptions in HTx patients.
